# Effects of GLP-1 on ovarian dysfunction in polycystic ovary syndrome: A protocol for systematic review and meta-analysis

**DOI:** 10.1097/MD.0000000000032312

**Published:** 2023-01-13

**Authors:** Caifeng Zhang, Dongling Yan, Xiaojing Wang, Dianchen Cheng

**Affiliations:** a Department of Gynaecology, Lanzhou Maternal and Child Health Hospital, Gansu, China; b Department of Anesthesiology, Lanzhou Maternal and Child Health Hospital, Gansu, China.

**Keywords:** 1, analysis, glucagon, like peptide, meta, ovarian dysfunction, polycystic ovary syndrome

## Abstract

Polycystic ovary syndrome (PCOS) is a frequent endocrine disorder in women, it is the principal cause of infertility and amenorrhea. Due to its high recurrence rate, poor prognosis and serious complications, more works on the research of PCOS are needed. Therefore, we performed a protocol for systematic review and meta-analysis to investigate the efficacy and safety of glucagon-like peptide-1 receptor agonists on ovarian dysfunction in PCOS.

**Methods:**

This study protocol has been registered in the PROSPERO and the registration number is CRD42020188247. The procedure of this protocol will be conducted according to the Preferred Reporting Item for Systematic Review and Meta-analysis Protocols guidance. A comprehensive search of several databases from 1966 to November 2022 will be conducted. The databases includes Ovid Medline In-Process & Other Non-Indexed Citations, Ovid MEDLINE, Ovid EMBASE, Ovid Cochrane Central Register of Controlled Trials, Ovid Cochrane Database of Systematic Reviews, and PubMed. The risk of bias of the included studies will be assessed using the Cochrane tool of risk of bias. All statistical analyses will be conducted using the software program Review Manager version 5.3.

**Results:**

The results of this systematic review will be published in a peer-reviewed journal.

**Conclusion:**

This systematic review will provide evidence to judge whether glucagon-like peptide-1 receptor agonist is superior to metformin in patients with PCOS.

## 1. Introduction

Polycystic ovary syndrome (PCOS) is 1 of the most common endocrine conditions, affecting 8 to 13% of reproductive aged women.^[1–3]^ Heterogeneous by nature, PCOS is defined by a combination of signs and symptoms of androgen excess and ovarian dysfunction in the absence of other specific diagnoses.^[4,5]^ Since PCOS affects many organ systems and has complex clinical presentation, it is recommended to treat the condition with a personalized approach that is based on individual manifestations.^[6]^ The recommended treatments for PCOS women, especially for PCOS patients with obesity, are lifestyle and nutrition interventions and weight loss.^[7–9]^ The treatment approach to PCOS generally focuses on goals for fertility or ovulation, bothersome hirsutism, and the screening and modification of common metabolic derangements.^[10]^ Reduction in body weight has been demonstrated to improve hyperandrogenism, reproductive function, and metabolic parameters such as hypertension, hyperlipidemia, and glycemic control in women with PCOS.^[11]^ The use of glucagon-like peptide-1 (GLP-1) receptor agonists offers a unique opportunity to address both excess body weight and glycemic control in 1 medical therapy.^[12,13]^

GLP-1 released from gut enteroendocrine cells controls meal-related glycemic excursions through augmentation of insulin and inhibition of glucagon secretion.^[14]^ GLP-1 also inhibits gastric emptying and food intake, actions maximizing nutrient absorption while limiting weight gain. GLP-1 receptor agonist therapy pharmacologically raises the available plasma GLP-1 to supraphysiologic levels. The GLP-1 receptor agonists have achieved remarkable weight reduction and abdominal fat loss in patients with type 2 diabetes, as well as in overweight/obese individuals and individuals with prediabetes.^[15]^ They have also been shown to promote lower fasting insulin levels and insulin resistance markers. These beneficial effects have been suggested to be particularly helpful in women with PCOS, while their possible role in the hypothalamic-pituitary-gonadal axis is under intense research. Therefore, we performed a protocol for systematic review and meta-analysis to investigate the efficacy and safety of GLP-1 receptor agonist on ovarian dysfunction in PCOS.

## 2. Methods

### 2.1. Registration

This study protocol has been registered in the PROSPERO and the registration number is CRD42020188247. The procedure of this protocol will be conducted according to the Preferred Reporting Item for Systematic Review and Meta-analysis Protocols guidance.^[16]^ No ethical statement will be required for this study because there is no direct involvement of human.

### 2.2. Inclusion and exclusion criteria

Eligible studies are accepted if they complied with the following inclusion criteria: Participants: patients with a diagnosis of PCOS; Intervention(s): the combination of GLP-1 receptor agonist and metformin are applied to PCOS patients; comparison(s): only metformin is used; Outcomes: changes in obesity, menstrual frequency, metabolic or endocrine parameters, and adverse events; Study design: randomized controlled trials with results published in English.

The exclusion criteria are as follows: insufficient outcome data; lack of a control group; case reports, comments or letters, biochemical trials, conference abstracts, reviews, and retrospective studies or prospective non-randomized studies.

### 2.3. Search methods

A comprehensive search of several databases from 1966 to November 2022 will be conducted. The databases includes Ovid Medline In-Process & Other Non-Indexed Citations, Ovid MEDLINE, Ovid EMBASE, Ovid Cochrane Central Register of Controlled Trials, Ovid Cochrane Database of Systematic Reviews, and PubMed. Search strategy for PubMed is shown in Table 1.

**Table 1:** Search strategy for PubMed.

#1 polycystic ovary syndrome [Title/Abstract] #2 Stein-Leventhal syndrome [Title/Abstract] #3 sclerocystic ovarian degeneration [Title/Abstract] #4 sclerocystic ovary syndrome [Title/Abstract]
#5 PCOS [Title/Abstract]
#6 or/#1–#5#7 glucagon-like peptide-1 [Title/Abstract]
#8 GLP-1 [Title/Abstract]
#9 glucagon-like peptide-1 receptor agonists [Title/Abstract]
#10 GLP-1 receptor agonists [Title/Abstract]
#11 GLP-1 RA [Title/Abstract]
#12 enterocretin [Title/Abstract]
#13 or/#7–#12#14 metformin [Title/Abstract]
#15 randomized controlled trial[Publication Type]
#16 randomly [Title/Abstract]
#17 randomized [Title/Abstract]
#18 or/#15–#17
#19 #6 and #13 and #14 and #18

### 2.4. Study selection

**Figure 1.**
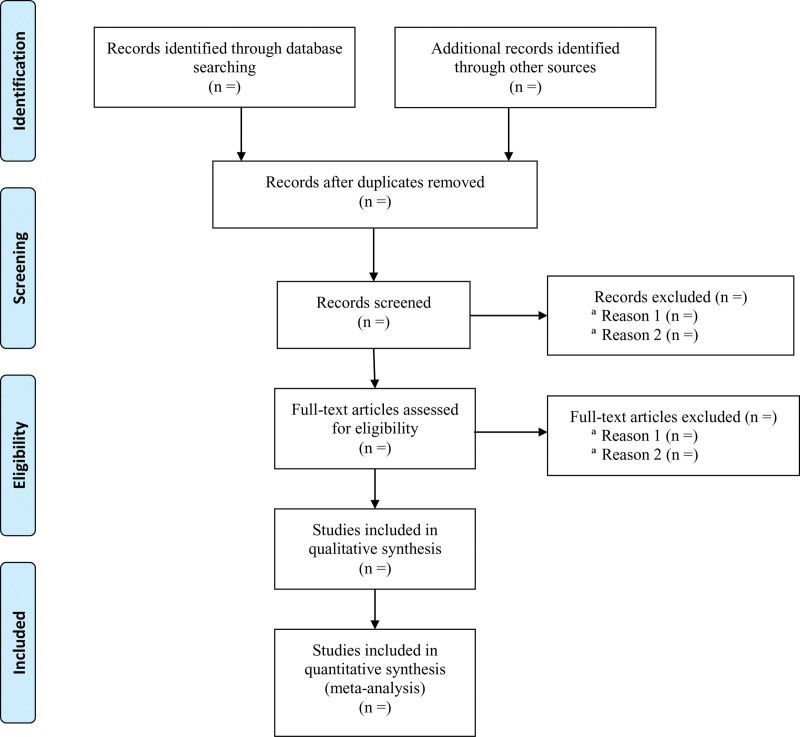
Preferred Reporting Items for Systematic reviews and Meta-Analyses (PRISMA) flow chart for the identification, inclusion and exclusion of studies.

We will export the identified records in databases into EndNote X9 software and use this to identify duplicates. After removing duplicates, the retrieved records will be checked independently by 2 reviewers, who will apply the eligibility criteria based on the title and abstract. Where a study is potentially eligible, the full text will be obtained and checked independently by 2 reviewers to identify the eligible studies. Any disagreements will be discussed and resolved in discussion with a third reviewer. Details of the selection procedure for the studies are shown in the PRISMA flow chart (Fig. 1).

### 2.5. Data extraction

Two review authors will independently extract the data and fill out the standard data extraction form, which includes study information such as the first author, publication year, title, journal name, research design, number of patients, inclusion criteria, interventions, control, treatment period, and outcome measures. Disagreements are resolved through discussion among all authors.

### 2.6. Risk of bias assessment

The risk of bias will be assessed independently by 2 authors using the Cochrane tool of risk of bias (V.5.1.0).^[17]^ The following items will be assessed: random sequence generation (selection bias), allocation concealment (selection bias), blinding (performance bias and detection bias), incomplete outcome data (attrition bias), selective outcome reporting (reporting bias), and other bias. The judgments of evaluated domains will include high, low, and unclear. Disagreements will be resolved by discussion by arbiter.

### 2.7. Data synthesis

Differences between the intervention and control groups will be assessed. Mean differences (MDs) with 95% confidence intervals (CIs) will be used to measure the effects of treatment for continuous data. We will convert other forms of data into MDs. For outcome variables on different scales, we will use standard MDs with 95% CIs. For dichotomous data, we will present the treatment effects as relative risks with 95% CIs, and other binary data will be converted into relative risk values. All statistical analyses will be conducted using the software program Review Manager version 5.3 (Copenhagen, The Nordic Cochrane Centre, the Cochrane Collaboration, 2014) for Windows. We will contact the corresponding authors of the studies with missing information to acquire and verify the data, whenever possible. When appropriate, we will pool the data across studies to conduct a meta-analysis using fixed or random effects.

### 2.8. Sensitivity analysis

Sensitivity analysis will be also applied to evaluate the robustness and reliability of the combined results of included studies. Methodological quality, heterogeneity, studies quality and sample characteristic will be considered.

### 2.9. Publications bias

We will conduct analysis of Egger publication bias plot and Begg funnel plot with pseudo 95% confidence limits to determine the publication bias in all the literature with sufficient studies (more than 10 trials).^[18]^

### 2.10. Grading quality of evidence

Furthermore, for grading the strength of the evidence for all outcomes from the included data, the Grading of Recommendations Assessment, Development and Evaluation method or an equivalent methodology will be clearly described and documented by 2 independent researchers.

## 3. Discussion

PCOS is a frequent endocrine disorder in women, it is the principal cause of infertility and amenorrhea.^[4,19]^ Due to its high recurrence rate, poor prognosis and serious complications, more works on the research of PCOS are needed. GLP-1 receptor agonists are a class of novel anti-diabetes agents and share similar effects of GLP-1, including glucose-dependent enhancement of insulin secretion and islet B cells proliferation. Previous studies have reported the use of GLP-1 receptor agonists in treating PCOS,^[13]^ however the results are limited by small sample size and low quality. This systematic review will provide current evidence from randomized controlled trials on the effectiveness and safety of GLP-1 receptor agonists for PCOS. These findings may help provide guidance to clinicians in the treatment of PCOS. The results of this report will be disseminated after peer review and publication.

## Author contributions

**Data curation:** Dongling Yan.

**Investigation:** Xiaojing Wang.

**Writing – original draft:** Caifeng Zhang.

**Writing – review & editing:** Dianchen Cheng.
